# Cognitive Impairment in Late-Life Depression: A Comparative Study of Healthy Older People, Late-Life Depression, and Mild Alzheimer's Disease Using Multivariate Base Rates of Low Scores

**DOI:** 10.3389/fpsyg.2021.724731

**Published:** 2021-10-05

**Authors:** Caroline Masse, Pierre Vandel, Géraldine Sylvestre, Nicolas Noiret, Djamila Bennabi, Frédéric Mauny, Marc Puyraveau, Yoan Barsznica, Jonathan Dartevelle, Agatha Meyer, Mickaël Binetruy, Marie Lavaux, Ilham Ryff, Julie Giustiniani, Eloi Magnin, Jean Galmiche, Emmanuel Haffen, Gilles Chopard

**Affiliations:** ^1^Department of Clinical Psychiatry, Besançon University Hospital, Besançon, France; ^2^Laboratory of Neurosciences and Cognitive Psychology, University of Bourgogne Franche-Comté, Besançon, France; ^3^Association for the Development of Applied Neuropsychology, Besançon, France; ^4^Clinical Investigation Center 1431-INSERM, Besançon University Hospital, Besançon, France; ^5^Department of Neurology, Memory Resource and Research Center (CM2R), Besançon University Hospital, Besançon, France; ^6^Research Centre on Cognition and Learning (CeRCA), UMR 7295 CNRS, University of Poitiers and University of Tours, Poitiers, France; ^7^Methodology Unit, uMETh, Clinical Investigation Center 1431-INSERM, Besançon, France; ^8^Laboratory of Chrono-Environnement, UMR 6249 CNRS, University of Bourgogne Franche-Comté, Besançon, France

**Keywords:** late-life depression, Alzheimer's disease, older people, base rates, low scores, false positive, misdiagnosis, cognitive impairment

## Abstract

Late-Life Depression (LLD) is often associated with cognitive impairment. However, distinction between cognitive impairment due to LLD and those due to normal aging or mild Alzheimer's Disease (AD) remain difficult. The aim of this study was to present and compare the multivariate base rates of low scores in LLD, mild AD, and healthy control groups on a battery of neuropsychological tests. Participants (ages 60–89) were 352 older healthy adults, 390 patients with LLD, and 234 patients with mild AD (i.e., MMSE ≥ 20). Multivariate base rates of low scores (i.e., ≤ 5th percentile) were calculated for each participant group within different cognitive domains (verbal episodic memory, executive skills, mental processing speed, constructional praxis, and language/semantic memory). Obtaining at least one low score was relatively common in healthy older people controls (from 9.4 to 17.6%), and may thus result in a large number of false positives. By contrast, having at least two low scores was unusual (from 0.3 to 4.6%) and seems to be a more reliable criterion for identifying cognitive impairment in LLD. Having at least three low memory scores was poorly associated with LLD (5.9%) compared to mild AD (76.1%) and may provide a useful way to differentiate between these two conditions [$\chi_{\left(1\right)}^{2}$ = 329.8, *p* < 0.001; Odds Ratio = 50.7, 95% CI = 38.2–77.5]. The multivariate base rate information about low scores in healthy older people and mild AD may help clinicians to identify cognitive impairments in LLD patients, improve the clinical decision-making, and target those who require regular cognitive and clinical follow-up.

## Introduction

Late-life depression (LLD), defined as a major depressive episode occurring in individuals 60 years or older (Bhalla and Butters, 2011; Koening et al., 2014), independent of age at onset (O'Hara et al., 2006), is a heterogeneous mood disorder that has been found to have negative impact effect on people's health-related quality of life (Kiosses et al., 2001). In a clinical setting, patients with LLD frequently report subjective cognitive complaints (Gonda et al., 2004) and may show cognitive impairment in several neuropsychological domains including, most notably, executive function (Lockwood et al., 2002; Dybedal et al., 2013), psychomotor speed (Butters et al., 2004; Bennabi et al., 2013), and episodic memory (Herrmann et al., 2007; Dybedal et al., 2013). Visuospatial ability (Butters et al., 2004), semantic memory, and oral language (O'Hara et al., 2006) have also been found to be impaired in patients with LLD.

However, the pattern of cognitive impairment in LLD remains unclear and varies across studies due to methodological factors such as differences in patients sampled or the variety of tools used to measure cognition (Jaeger et al., 2006; Godin et al., 2007). In addition, most studies available had modest sample sizes (Dybedal et al., 2013; Roca et al., 2015) or reported cognitive functioning in terms of group means in a case-control design (Herrmann et al., 2007; Korsnes and Ulstein, 2014) that may obscure the degree of cognitive impairment in LLD (Gualtieri and Morgan, 2008; Iverson et al., 2011). An alternative approach for interpretation pattern of cognitive impairment is to determine the prevalence of low test-scores within a neuropsychological battery (Gualtieri and Morgan, 2008; Iverson et al., 2008, 2011; Brooks et al., 2011; Holdnack et al., 2017).

However, identification of cognitive impairment in patients with LLD remains difficult without taking into account normal cognitive variability in the healthy older population. Indeed, a great deal of research has demonstrated that the presence of one or more low scores was common among healthy older adults when multiple measures were considered simultaneously rather than in isolation (Palmer et al., 1998; Brooks et al., 2012; Gunner et al., 2012). For example, when considering performance across all subtests from the CERAD (Consortium to Establish a Registry for Alzheimer's Disease) neuropsychological assessment battery, 60.6% of the healthy older participants (aged 49–92 years) obtained one or more score at or below the 10^th^ percentile (Mistridis et al., 2015). Using the RAPID (Regional Network for Diagnostic Aid and Management of Patients with Cognitive Impairment) neuropsychological battery, we previously found that 40.1% of the normative sample (individuals aged 50–89 years) obtained one or more test scores at or below the 5th percentile (Sylvestre et al., 2017). These findings suggest that isolated low scores may be erroneously considered impaired performances and lead to a false positive interpretation (Schretlen et al., 2008).

Many other studies (Palmer et al., 1998; Schretlen et al., 2008; Brooks et al., 2009a; Gunner et al., 2012; Oltra-Cucarella et al., 2019) also highlighted the need to consider base rates of low scores in healthy older individuals (i.e., false positive) in drawing inferences from low-test performances in patients, including those with neurological or psychiatric disorders (Brooks et al., 2009b; Karr et al., 2017). Indeed, the presence of cognitive impairment in patients with LLD may be sometimes due to the development of incipient dementia such Alzheimer's disease (AD) (Rushing et al., 2014, Ly et al., 2021). In clinical practice, the distinction of cognitive impairment related to LLD from those associated with early stage of AD is particularly difficult (Swainson et al., 2001; Foldi et al., 2003; Mazur-Mosiewicz et al., 2011; Gasser et al., 2018; Lanza et al., 2020a) and may lead to misdiagnosis in the older population (Rotomskis et al., 2015).

In view of the above mentioned, the aim of the present study was to describe the cognitive performance in patients with LLD in comparison with those with mild AD and a healthy control group. To achieve this aim, this study examines and presents the multivariate base rates of low scores on the RAPID battery in patients with LLD, mild AD, and older healthy adults. It is hypothesized that patients would have more low scores on the RAPID test battery compared with healthy older people controls. It is further hypothesized that the base rate information could help to distinguish cognitive impairments associated with LLD from those observed with mild AD or normal aging.

## Methods

### Participants

The study sample consisted of a healthy older people control group and two clinical groups including patients with LLD and patients with AD.

The healthy older people control group were subjects drawn from the RAPID normative sample whose neuropsychological data had been initially collected previously (Ferreira et al., 2010). The design and methods for the RAPID normative data have been described in detail elsewhere (Ferreira et al., 2010). Briefly, to be included in the study, subjects had to speak and comprehend French, have no visual deficits or hearing loss that could interfere with the administration of neuropsychological testing, and had to live independently according to the “instrumental activities of daily living” (Lawton and Brody, 1969). Any participant with a previous medical history of a neurological disease (i.e., head trauma, stroke, dementia, Parkinson's disease, epilepsy, or brain tumor) or a psychiatric disorder (i.e., major depression, schizophrenia, bipolar disorder) was not included. As older adults represent the population of interest, individuals under 60 years of age were not included, thus the final group consisted of 352 healthy older people controls aged 60–89 years.

The clinical groups were convenience samples of patients with LLD or with AD. All patients had been referred to one of the nine inpatient or outpatient local memory consultations centers in the Franche-Comté Region (France) between 2008 and 2016. Cognitive data on all patients were retrospectively obtained from the database Rapid-Fr network (Bereau et al., 2015). The aim of this health network, financed by the French government, is to coordinate memory consultations in the geographical area of Franche-Comté (France). All patients were between 60 and 89 years old. Exclusion criteria were determined by reviewing the medical records.

Patients with LLD met the diagnostic criteria of major depression disorder according to the Diagnostic and Statistical Manual of Mental Disorders, Fourth Edition (DMS-IV) (American Psychiatric Association, 1994) and after a comprehensive clinical interview by a psychiatrist. All patients were or had been recently hospitalized and all were on medication. Exclusion criteria were: psychotic features, schizophrenia, bipolar disorder, current, or past history of neurological disease (e.g. dementia, head trauma, Parkinson's disease, epilepsy) substance abuse or a history of electroconvulsive treatment within the past 12 weeks. The final LLD sample included 390 patients.

The AD patients met National Institute of Neurological and Communicative Disorders and Stroke Alzheimer's Disease and Related Disorders Association for probable AD (NINCDS-ADRDA criteria) (McKhann et al., 1984) after full workup including neurologic, cognitive, imaging, and laboratory tests. Exclusion criteria were: concurrent neurological disorder, an acute stroke and a Fazekas score > 2 to avoid mixtures of vascular and neurodegenerative processes (Fazekas et al., 1993). Given that depressive symptoms are commonly reported in patients with AD (20–30%) (Lyketsos et al., 2002; Zubenko et al., 2003) we did not exclude them from this study (Foldi et al., 2003). Because the differentiation between of depression from AD in older age is more difficult in the earliest stages of AD (Rotomskis et al., 2015), only mild AD patients (i.e., MMSE ≥ 20) were included in the current study. The final AD sample included 234 patients.

### Neuropsychological Assessment

The RAPID battery was developed by a clinical research consortium in the Franche-Comté region (France) using the same standardized evaluation. The purpose of this consortium was to facilitate patient care management throughout the Franche-Comté region using a common neuropsychological battery of tests and software database (Bereau et al., 2015). All the tests of this battery were initially normed based on the same normative sample for a broad range of ages and educational levels (Ferreira et al., 2010).

All participants were administered the RAPID battery by trained neuropsychologists. The full test battery takes approximately 60 min to complete and includes nine tests that yield 13 primary independent scores measuring five cognitive domains: verbal episodic memory, executive skills, mental processing speed, constructional praxis, and language/semantic memory. The tests administered for each domain are described below.

### Assessment of Verbal Episodic Memory

The Free and Cued Selective Reminding Test (FCSRT, Grober and Buschke, 1987). We used a french adaptation by Van der Linden et al. (2004). This test assesses the ability to learn a 16 written word list that refers to 16 semantic categories. After an encoding phase, participants have to perform three successive recall trials separated by an interfering task. Each trial includes two parts. First, each participant had to freely recall as many items as possible. Next, an orally presented semantic category (e.g., what was the name of the fish?) was provided for the words that were not spontaneously retrieved by the participants. The total free recall score (from 0 to 48) (i.e., participants are asked to retrieve the words spontaneously) and the total cued recall score (i.e., participants are asked to retrieve the words with the help of a semantic cue) were considered.

The Memory Impairment Screen (MIS, Buschke et al., 1999): a French version of the MIS was used (Chopard et al., 2007). The MIS is a 4-min, four-item delayed free- and cued-recall memory test. After an interfering task lasting at least 2 min, the subjects are invited to reproduce the words learned in any order over 20 s (free recall). A cued recall is proposed for any words not mentioned using free recall. For each word, the score for a correct answer is two points for free recall and one point for cued recall. The total MIS score ranged from 0 to 8 was considered.

The word delayed recall of the Mini-Mental State Examination (MMSE) (Folstein et al., 1975): the MMSE is a global cognitive efficiency evaluation through use of 30 items to assess orientation, calculation, memory, language, and visio-constructional abilities. In this study, a consensual French version developed by GRECO (Group of Research and Cognitive Assessments) was used (Kalafat et al., 2003). The delayed recall of the three words (from 0 to 3) was considered.

### Assessment of Executive Functions

The Trail Making Test, part B (TMTB, Reitan, 1958): participants are required to connect numbers and letters alternatively as quickly as possible. The total time to complete the TMT part B was considered as well as the total number of errors (i.e., sequential errors and/or perseverative errors).

The Isaacs Set Test (IST, Isaacs and Kennie, 1973): the IST is a 1-min verbal category fluency task. The subject has to orally produce as many words as possible in 15 s for each of the following categories: colors, animals, fruit, and cities. The total number of items named was considered (any repeated words or intrusions are not included).

### Assessment of Processing Speed

The Trail Making Test part A (TMTA, Reitan, 1958): participants are required to connect with lines 25 circles numbered from 1 to 25 as quickly as possible. The total time to complete the TMTA was considered.

The Crossing-Off Test (COT, Botwinick and Storandt, 1973): the COT is a psychomotor task in which the participants have to make a slash mark through a set of 96 horizontal lines, as quickly as possible. There are eight lines per row, and individuals cross out the lines from left to right, one row after another. This test is scored as the number of lines (96) crossed out per second (*t*) times 100 (Index of rapidity = 96/*t* × 100).

### Assessment of Constructional Praxis

Copy of triangles from the Battery of Cognitive Efficacy (BEC 96): the copy of the triangles of the cognitive assessment battery test in 96 items (Signoret et al., 1998) is a test in which the subject must copy a geometric figure according to a model that is presented. This figure is made up of a set of three intersecting triangles. The total final score, which varies from 0 to 6, was considered.

The pentagon copy of MMSE (Folstein et al., 1975): copying the overlapping pentagons is a standard sub-item of the MMSE. It was scored dichotomously as either correct (1) or incorrect (0).

### Assessment of Language/Semantic Memory

Oral name of images: oral denomination of 30 images (D030, Ferreira et al., 2010) is a test in which the subject must name 10 animals, 10 objects, and 10 actions. These images are from the BEC 96 (Signoret et al., 1998), the test D080 (Metz-Lutz et al., 1991), and the Montreal-Toulouse test of aphasia review protocol (Nespoulous et al., 1992). The overall score, made up of the total number of correct answers (from 0 to 30), was retained.

Categorical Matching: the Categorical Matching Test (Ferreira et al., 2010) assesses the subject's ability to perform semantic associations in the presence of distractors. The subject must designate one of three visual items, the one that is semantically connected to the target item. The total score of correct answers, which ranges from 0 to 10, was considered.

It should be noted that in our study sample the diagnosis of AD was not entirely blind to cognitive performance and therefore raises the question of a partial circularity. However, the construct of base rates of low scores was only conceived after the RAPID data were collected. Moreover, other tests such as the California Verbal Learning Test (Delis et al., 1987), the Wechsler Memory Scale (Wechsler, 2001, 2012), the Delayed Matching-to-sample Task (Barbeau et al., 2004; Rullier et al., 2014), the copy, immediate recall, and delayed recall conditions of the Rey-Osterrieth Complex Figure (Osterrieth, 1944; Rey, 1959; Meyers and Meyers, 1994), the Stroop test (Stroop, 1935), the GRECO neuropsychological semantic battery (Merck et al., 2011), the phonemic and semantic verbal fluency tests (Cardebat et al., 1995), or the oral denomination of 80 images (Metz-Lutz et al., 1991) in addition to the neuropsychological RAPID test Battery, have also been used to further assess the cognitive functioning contributing to limit the risk of circularity.

## Data Analysis

Given that most of the primary measures of the RAPID test battery did not meet the normal distribution criteria (Ferreira et al., 2010), a low score was defined as less than or equal to the 5th percentile, which is a common psychometric criterion used in clinical practice to determine “cognitive impairment” (Lezak et al., 2004). This cut-off score was also included in previous multivariate base rate analyses (Brooks et al., 2009a,b; Iverson et al., 2011; Ivins et al., 2015; Holdnack et al., 2017; Karr et al., 2017; Rivera et al., 2019).

The 5th percentile cut-off values for the 13 primary measures of the RAPID test battery were calculated in a previous study (Sylvestre et al., 2017) from the normative sample of older adults (Ferreira et al., 2010) based on three age categories (ranges 60–69, 70–79, and 80–89 years) and three educational levels: high level (i.e., >11 years of education), middle level (i.e., 8–11 years of education), or the lowest educational level (i.e., <8 years of education).

As a reminder, to estimate the variability of the number of low scores in each of the RAPID primary measures, 1,000 bootstrap replicates were computed (Efron, 1992). Rather than limiting ourselves to an observed value for a small number of subjects, we wanted to add an uncertainty factor to our estimation. With the “bootstrapping” technique, we were able to calculate the variability of an estimation as the probability laws of this was unknown.

The multivariate base rates of low scores on the RAPID battery for the healthy older people controls, LLD, and AD group were determined by simultaneously examining the performance of 13 primary measures. For each subject, the total number of low scores (i.e., at or below the 5th percentile) was calculated across all primary measures. The multivariate base rates of low scores were further calculated as cumulative percentages for each of the three groups within different cognitive domains: verbal episodic memory, executive skills, mental processing speed, constructional praxis, and language/semantic memory. A Chi-square test was used to compare relevant group frequencies generated by cross tabulation.

## Results

The demographic characteristics for each of the three groups are shown in Table 1.

**Table 1:** Demographic characteristics of the samples.

	**HC**	**LLD**	**Mild AD**
Sample size	352	390	234
Mean age (SD)	72.9 (7.7)	73.1 (7.7)	78.9 (6.1)
Age range	60–89	60–89	60–89
Education level			
High (%)	16.1	13.4	23.5
Middle (%)	32.5	32	28.2
Low (%)	51.4	54.6	48.3
Male/Female (%)	37/63	29/71	36/64

*HC, healthy controls; LLD, late life depression; AD, Alzheimer's disease; SD, standard deviation. High (i.e., >11 years of education), Middle (i.e., 8–11 years of education), Low (<8 years of education)*.

The base rates of low scores within specific cognitive domains are presented in Table 2. As seen in Table 2, it is common (between 9.4 and 17.6%) for healthy older people to obtain at least one low score (i.e., ≥1). For example, they were 17.6 and 16.5% for verbal memory and executive function, respectively. The prevalence of having at least two low scores (i.e., ≥2) dramatically decreased and varied from 0.3 to 4.6%. For example, they were 4 and 4.6% for verbal memory and executive function, respectively. A great percentage of patients obtained at least one low score: between 12.8 and 64.1% for LLD, between 11.2 and 95.7% for mild AD. The prevalence of having at least two low scores varied from 1.8 to 48.1% for LLD and from 0.8 to 87.3% for mild AD.

**Table 2:** Base rates of low scores within specific cognitive domains of healthy controls and patients with late life depression and mild Alzheimer's Disease.

	**Number of low scores**				
	**0**	**≥1**	**≥2**	**≥3**	**4**
Verbal memory (HC)	82.4	17.6	4	1.7	0.6
Verbal memory (LLD)	48.8	51.2	21	5.9	1
Verbal memory (AD)	4.3	95.7	87.3	76.1	48.3
Executive skills (HC)	83.5	16.5	4.6	0.3	n/a
Executive skills (LLD)	35.9	64.1	48.1	17.9	n/a
Executive skills (AD)	26.1	73.9	50	18.4	n/a
Mental proc. speed (HC)	90.5	9.5	1.1	n/a	n/a
Mental proc. speed (LLD)	63.7	36.3	16.1	n/a	n/a
Mental proc. speed (AD)	75.2	24.8	6	n/a	n/a
Constr. praxis (HC)	90.3	9.4	0.3	n/a	n/a
Constr. praxis (LLD)	87.2	12.8	3.1	n/a	n/a
Constr. praxis (AD)	88	11.2	0.8	n/a	n/a
Language/Semantic (HC)	88.1	11.9	1.4	n/a	n/a
Language/Semantic (LLD)	85.4	14.6	1.8	n/a	n/a
Language/Semantic (AD)	66.3	33.7	3.8	n/a	n/a

*HC, healthy older people controls; LLD, late life depression; AD: Alzheimer's disease; Cognitive domains defined as follows: verbal memory: MIS, memory impairment screen; MMSE-word recall, mini-mental state examination; FCSRT, Free and cued selective reminding test; free recall and cued recall; executive skills: TMT B, trail making test; time and number of errors; IST, Isaacs set test; processing speed: COT, crossing-off test; TMT, trail making test; A-time; constructional praxis: MMSE-copy, BEC 96 figures-copy; language and semantic memory: categorical matching, naming test, n/a: not applicable*.

When comparing the cumulative percentages of participants in LLD and healthy older people control group, a larger percentage of patients with LLD had two or more low verbal memory scores than healthy controls (21 vs. 4%) [$\chi_{\left(1\right)}^{2}$ = 47.74, *p* < 0.001; Odds Ratio = 6.4, 95% CI = 3.8–10.9]. In the executive domain, a much higher percentage was found in patients with LLD (48.1%) than in healthy older people controls (4.6%) [$\chi_{\left(1\right)}^{2}$ = 175.38, *p* < 0.001; Odds Ratio = 19.4, 95% CI = 12.5–30]. Patients with LLD are more likely to obtain two or more low processing speed scores (16.1%) than healthy older people controls (1.1%) [$\chi_{\left(1\right)}^{2}$ = 50.80, *p* < 0.001; Odds Ratio = 16.8, 95% CI = 7.7–36.4]. For the constructional praxis, having two low scores occurred in 3.1% of the LLD patients and 0.3% of the healthy controls [$\chi_{\left(1\right)}^{2}$ = 8.4, *p* < 0.01; Odds Ratio = 11.1, 95% CI = 2.2–57]. For the language/semantic memory domain the percentages look very similar (1.8 vs. 1.4%) in both groups (χ^2^ = 0.16, *p* = 0.68; Odds Ratio = 1.3, 95% CI = 0.4–4).

When comparing the cumulative percentages of participants in LLD and mild AD group (mean MMSE = 23.1 ± 2.2), a larger percentage of patients with mild AD had two or more low verbal memory scores than LLD (87.3 vs. 21%) [$\chi_{\left(1\right)}^{2}$ = 257.81, *p* < 0.001; Odds Ratio = 25.5, 95% CI = 1.7–37.5]. In the executive domain, the percentages look very similar in both groups (48.1 vs. 50%) [$\chi_{\left(1\right)}^{2}$ = 0.24, *p* = 0.62; Odds Ratio = 1.1, 95% CI = 0.8–1.5]. Patients with LLD are more likely to obtain two low processing speed scores than those with mild AD (16.1 vs. 6%) [$\chi_{\left(1\right)}^{2}$ = 13.44, *p* < 0.001; Odds Ratio = 3.0, 95% CI = 1.7–5.3]. For the constructional praxis [$\chi_{\left(1\right)}^{2}$ = 3.29, *p* = 0.07; Odds Ratio = 0.27, 95% CI = 0.06–1.2] and language/sematic memory domain [$\chi_{\left(1\right)}^{2}$ = 2.46, *p* = 0.11; Odds Ratio = 2.19, 95% CI = 0.8–5.8], there was no significant statistical difference.

Having three low scores in the executive domain, was found in the same proportion for both LLD (17.9%) and AD (18.4%) group (χ^2^ = 0.02, *p* = 0.89; Odds Ratio = 1.03, 95% CI = 0.7–1.5). A larger proportion of patients with mild AD (76.1%) than patients with LLD (5.9%) had three or more low scores (i.e., ≥3) in the verbal memory domain [$\chi_{\left(1\right)}^{2}$ = 329.8, *p* < 0.001; Odds Ratio = 50.7, 95% CI = 38.2–77.5].

## Discussion

The results of the present study reiterate that a substantial proportion of healthy older individuals obtained one or more low scores on the RAPID battery even at a stringent cut-off value (i.e., at or below the 5th percentile). They are consistent with previously reported studies on other neuropsychological test batteries (Heaton, 1991; Palmer et al., 1998; Brooks et al., 2009a, 2011; Karr et al., 2017) and confirmed that low scores are common in healthy older people when multiple test scores are simultaneously analyzed across a battery. This implies that isolated low scores obtained by an individual across multiple tests are not necessarily pathological or indicative of truly impaired functioning (Schretlen et al., 2008; Binder et al., 2009; Sylvestre et al., 2017). Thus, our data showed that from 9.4 to 17.6% of healthy older people obtained at least one low score on either measure in each cognitive domain. These findings suggest that an isolated low score may result in a large number of false positives and may have little relevance in clinical evaluation (Palmer et al., 1998; Lezak et al., 2004). Indeed, if one or more low scores at or below the 5th percentile is used as a criterion for identifying memory, executive or speed processing impairment on the RAPID battery, it can be noted that, respectively, 17.6, 16.5, and 9.5% of healthy controls obtained such a result (i.e., a potential false positive). It is also important to emphasize that these percentages are much higher than the theoretical base rate of ≤ 5% when interpreting a single score in isolation (Brooks et al., 2013; Karr et al., 2017; Rivera et al., 2019). By contrast, having two or more low scores could be used as a more confident criterion since this pattern of results was uncommon in healthy older people controls (from 0.3 to 4.6%). This information on the base rate of low scores among healthy older adults is important since it may help clinicians to reduce the likelihood of misdiagnosing cognitive impairment among older depressed patients.

Indeed, many studies have already shown poor performance in verbal memory, executive function and processing speed in LLD (Baudic et al., 2004; Elderkin-Thompson et al., 2006; Dybedal et al., 2013). However, the majority of them reported results derived from comparisons of mean groups (i.e., average performance) in a case-control design, which may obscure the importance of cognitive impairment in patients with LLD (Gualtieri and Morgan, 2008; Iverson et al., 2011). It should be emphasized that a significant group difference does not inform about the proportion of patients who have cognitive impairment and those who have no cognitive impairment (Gualtieri and Morgan, 2008). This suggests that the average-to-average method is not sufficiently reliable to characterize the level of cognitive functioning in different samples of individuals (Ivins et al., 2015). From this perspective, presenting data in terms of frequency of low scores could be more informative and useful in clinical practice (Chelune, 2010; Iverson and Brooks, 2011; Ivins et al., 2015).

To the best of our knowledge, very few studies provided information about the prevalence of low scores for different cognitive domains in patients with LLD. One study (Dybedal et al., 2013) found that up to 50% of patients with LLD aged 60–85 years obtained a low score in at least one cognitive domain compared to 20% of the controls. Another study (Butters et al., 2004) showed that 61% of depressed patients aged 60 years and older had a low score in at least one domain compared to 32.5% of the controls. A recent research (Lanza et al., 2020b) found about one-third of depressed patients aged 54–81 years were below normal in executive function and about two out of three patients were below normal in memory function. However, in these researches, a low score was defined using the less stringent cut-off values (i.e., below the 10^th^ percentile or mean minus 1.5 standard deviation) of a very small control group (*n* = 18, *n* = 40, and *n* = 40, respectively) and not from large normative data stratified by age and education.

The comparison of the base rates of low scores in the LLD patients with that of mild AD showed a great overlap in the executive function. This result is in accordance with previous works and confirmed that tests of executive functions do not allow distinguish these two disorders (Swainson et al., 2001; Rushing et al., 2014). Executive dysfunction is common in LLD (Lockwood et al., 2002; Alexopoulos, 2003; Baudic et al., 2004; Herrmann et al., 2007; Dybedal et al., 2013) and may be broadly related to a slowed information processing speed (Butters et al., 2004) and frontostriatal circuit disorders caused by vascular lesions (Alexopoulos, 2006, Butters et al., 2008). In addition, executive dysfunction may also be secondary to a lack of motivation (Scheurich et al., 2008) or a decrease in effortful attention capacity (Elliot, 1998; Royall et al., 2012). The presence of executive impairment has also been demonstrated in the mild stages of AD in inhibition, task-switching, planning, flexibility, and concurrent manipulation of information (Colette et al., 1998; Perry et al., 2000; Stokholm et al., 2006; Godefroy et al., 2014).

This study also shows that a great percentage (51.2%) of individuals with LLD obtained at least one low memory score (see Table 2). Episodic verbal memory has been found to be impaired in older people with depression (Butters et al., 2004; Sheline et al., 2006; Lamar et al., 2012). It has been argued that memory impairment in LLD may be related to executive dysfunction (Fossati et al., 2002; Herrmann et al., 2007; Lamar et al., 2012). In addition, depressed patients may show a lack of effortful attention that may contribute to reduced memory performance (Hasher and Zacks, 1979). This view is consistent with the fact that depressed patients have more difficulty with tasks that require effortful information processing than those requiring automatic information processing (Hasher and Zacks, 1979; Hammar, 2003; Hammar et al., 2003). Our results are consistent with this and showed that in our LLD patient sample, the frequency of having a low score on the free recall of the FCSRT (25%) and of the MMSE (34%) (high-demanding tasks) was higher than on the cued recall of the FCSRT (12%) or the MIS (10%) that require much fewer attention resources. This result may reflect difficulty in retrieving information during effortful memory tasks in LLD (Alexopoulos, 2001).

It is also worth noting that a great proportion (76.1%) of individuals with mild AD obtained 3 or 4 low memory scores whereas this pattern of performance was uncommon (5.9%) in LLD patients. This result may be explained by the fact that AD patients are impaired on all memory tasks including effortful or automatic process (Leyhe et al., 2017). Thus, it has been suggested that cued recall could allow to differentiate between poor memory due to depression and those related to AD since it was expected to minimize the decreased attention resources or lack of strategies found most often in the patients with depression (Ivaniou et al., 2005; Dierckx et al., 2007). However, in the present study, if only 12% of LLD had a low performance on the cued recall of the FCSRT, 66% of mild AD obtained such a pattern showing that this index of measure is moderately sensitive to minimal impairment at a mild stage of the disease. Conversely, a greater percentage of mild AD obtained a low free recall at the FCSRT (84%) but this measure lacks of specificity since 25% of LLD patients (i.e., false positive) obtained such a score, demonstrating a great overlap of this measure for differentiating these two conditions. Our findings thus suggest that using a pattern of low scores on multiple memory measures seems to be able to distinguish patients with LLD from patients with mild AD. They are not surprising and consistent with previous studies reported that memory tasks have relatively high power to discriminate AD and older depressed patients. However, these studies have focused on a single or overall cognitive composite score and were carried out in small samples size which could result in a probable lack of statistical power (Swainson et al., 2001; Foldi et al., 2003; Künig et al., 2006; Dierckx et al., 2007, 2011; Federico et al., 2008; Contador et al., 2010; Para et al., 2010; Croisile et al., 2011; Mazur-Mosiewicz et al., 2011; Rotomskis et al., 2015).

The present study has several limitations. First, as in some previous works (Gualtieri and Morgan, 2008; Iverson et al., 2011), our LLD group was a convenience sample: the intensity of depression, age of onset or number of prior episodes had not been recorded in the RAPID database. Nevertheless, the great majority of LLD patients were or had been hospitalized for major depression, and all were in regular contact with their psychiatrist, meaning that they had probably moderate or severe depression rather than a mild form of depression. Furthermore, it has been shown (Purcel, 1997) that in-patients performed worse on cognitive tests than the out-patients, and thereby may limit the generalizability of our study results. In the future, it would be interesting to collect this information in order to better characterize the pattern of cognitive impairment of this condition. Second, our study did not take into account pharmacological treatment that could affect cognitive performance in LLD patients. Indeed, all depressed patients were being treated with antidepressants at the time of neuropsychological testing, which could affect the cognitive performance of LLD patients. Non-tricyclic antidepressants are known to improve memory performance (Haynes et al., 2004; McIntyre et al., 2014), whereas those with anticholinergic properties have been found to impair memory performance (Thompson and Trimble, 1982). Third, no information were recorded in the RAPID database about vascular factors in patients with LLD. Many studies have revealed a strong association between LLD, cognitive impairment, cerebrovascular disease, and poor cognitive outcomes, including progressive dementia, especially AD (Butters et al., 2008). Further assessment of the impact on the possible role of vascular comorbidity in the prevalence of low cognitive scores in LLD will be needed. In addition, as for LLD patients, we also used a convenience sample of patients with AD that may not be representative of community-dwelling adults. Thus, the findings of this paper should be validated using a randomly recruited, nationally, representative samples. Lastly, the ecological validity of the RAPID battery has never been examined in community-dwelling older adults. However, it is worth noting that norms of the RAPID battery tests for older adults were obtained from the same sample, in testing conditions as close as possible as those of a patient referred to a neuropsychologist, i.e., with the same tests, in the same order and time of administration.

However, this study has the advantage of showing results from a large sample (742 participants), thereby making it possible to present the data in terms of frequency of low scores (Gualtieri and Morgan, 2008). Furthermore, the 5th percentile cut-off values used in this study to define a low score were determined from age-and education-adjusted normative data (Sylvestre et al., 2017) that help to minimize over-or underestimation of impairment due to these two potential confounding variables (Potter and Steffens, 2007).

Furthermore, it is also important to remember that the base rates of low scores are expected to increase both as the number of tests increases and with a more lenient cut-off (Schretlen et al., 2008; Binder et al., 2009; Iverson and Brooks, 2011). This means that higher rates of false-positive low scores would have been reported in our healthy older people group if we had used a less stringent cut-off value (such as the 16th percentile) or a battery including a higher number of tests for each cognitive domain (Oltra-Cucarella et al., 2019). This implies that a higher number of low test-scores should be used as a reliable criterion for identifying (Mistridis et al., 2015) or predicting (Bradfield et al., 2020) cognitive impairment. This emphasizes the need for clinicians to interpret isolated low scores with even greater caution when using a large number of tests with a less conservative cut-off.

The findings of the present study may have potential implications for clinical practice: for each cognitive domain, having at least one low score on the RAPID battery was common among our healthy older adult sample and cannot therefore be used to support the presence of cognitive impairment in patients with LLD (i.e., high risk of false positive). However, having at least two low scores seems to be a more valuable criterion for identifying cognitive impairment in LLD, given its low positive rates in healthy older people. The base rate of low scores could thus provide adjunctive information in the clinical assessment of patients with LLD. It could for instance help to target patients with LLD who exhibit more extensive executive dysfunction and maybe at higher risk of poor outcomes (Koening et al., 2014) since this is known to be associated with poor antidepressant response (Potter et al., 2004; Alexopoulos et al., 2005; Story et al., 2008) and lower remission rates (Potter et al., 2004; Sheline et al., 2006). More studies are needed to explain the relationship between executive dysfunction and poor antidepressant response in LLD (Pimontel et al., 2016). Moreover, it could be interesting to combine many factors (clinical, cognitive, imaging, genetic, and so on) in order to better determine the risk of non-remission to antidepressants (Masse-Sibille et al., 2018).

In the verbal memory domain, the presence of three or more low memory scores may help establish whether the memory impairment is secondary to geriatric depression or to neurodegenerative process such as AD. This pattern of low scores could also be used as a red flag for memory impairment and should encourage the practitioner to target the LLD patient's follow-up. This point is relevant since LLD patients with poor memory performance have an increased risk of developing AD (Diniz et al., 2013; Kaup et al., 2016). It is an important challenge in psychiatry and neurology (Künig et al., 2006; Leyhe et al., 2017; Liguori et al., 2018) from a prognostic and therapeutic point of view (Birrer and Verumi, 2004; Mazur-Mosiewicz et al., 2011; Rotomskis et al., 2015; Gasser et al., 2018). However, future longitudinal research should consider estimating whether the base rates analyzing low memory scores by using different cut-offs, could have a useful predictive value for people with LLD.

In conclusion, the present study highlights the importance of knowing the base rates of low scores across a battery of tests in healthy older adults and mild AD for detecting the presence of cognitive impairment in older adults with depression. This base rate information may help reduce the likelihood of misdiagnosing cognitive impairments, improve the clinical decision-making and target LLD patients who require careful and regular cognitive follow up.

## Data Availability Statement

The original contributions presented in the study are included in the article, further inquiries can be directed to the corresponding author.

## Ethics Statement

The studies involving human participants were reviewed and approved by The ethics committee of Besançon university hospital. The patients/participants provided their written informed consent to participate in this study. The RAPID database was validated by an ethics committee: CNIL (French CNIL: 892143). All patients in the memory consultation center receive oral and written information on the possible use of their data for research purposes.

## Author Contributions

CM: analysis and interpretation of data, preparation of manuscript, and revising the manuscript. PV, DB, FM, MP, JGa, EM, JGi, JD, NN, YB, MB, and EH: revising the manuscript. GS: acquisition of subjects and data and revising the manuscript. AM, ML, MB, and IR: acquisition of subjects and data. GC: study concept and design, acquisition of subjects and data, analysis and interpretation of data, preparation of manuscript, and revising the manuscript. All authors contributed to the article and approved the submitted version.

## Conflict of Interest

The authors declare that the research was conducted in the absence of any commercial or financial relationships that could be construed as a potential conflict of interest.

## Publisher's Note

All claims expressed in this article are solely those of the authors and do not necessarily represent those of their affiliated organizations, or those of the publisher, the editors and the reviewers. Any product that may be evaluated in this article, or claim that may be made by its manufacturer, is not guaranteed or endorsed by the publisher.

## Abbreviations

AD: Alzheimer's disease
BEC 96: battery of cognitive efficacy
CERAD: consortium to establish a registry for Alzheimer's disease
COT: crossing-off test
DO30: denomination of 30 images
FCSRT: free and cued selective reminding test
GRECO: group of research and cognitive assessments
IST: Isaacs set test
LLD: late-life depression
MIS: memory impairment screen
MMSE: mini-mental state examination
NINCDS-ADRDA: National Institute of Neurological and Communicative Disorders and Stroke Alzheimer's Disease and Related Disorders Association for probable AD
RAPID: regional network for diagnostic aid and management of patients with cognitive impairment
TMTA: trail making test, part A
TMTB: trail making test, part B.
